# High-SNP-density genes and functional pathway analysis reveal key regulators of feed conversion ratio in broiler chickens

**DOI:** 10.1016/j.jgeb.2026.100809

**Published:** 2026-09-01

**Authors:** Heydar Ghiasi, Navid Ghavi Hossein-Zadeh

**Affiliations:** a Department of Animal Science, Faculty of Agricultural Science, Payame Noor University, Tehran 19395-4697, Iran; b Department of Animal Science, Faculty of Agricultural Sciences, University of Guilan, Rasht, Iran

**Keywords:** Feed conversion ratio, SNP annotation, Broiler chicken, Transcription factor motifs, Functional enrichment, VEP, *ST3GAL6*, *DGKZ*

## Abstract

Feed conversion ratio (FCR) is a critical economic trait in broiler production, influencing both productivity and sustainability. In this study, an initial set of 2272 peak SNP records associated with FCR was retrieved from the Animal QTL Database and, following filtering and removal of duplicate/redundant records, 513 unique, non-redundant SNPs were retained for functional annotation using the Ensembl Variant Effect Predictor (VEP). These SNPs were mapped to 543 genes, allowing a detailed analysis of their genomic distribution and functional effects. The majority of SNPs were intron variants (54.0%), followed by downstream (17.7%), upstream (10.2%), and synonymous variants (6.5%). Missense variants (1.4%) were identified in biologically relevant genes such as *ST3GAL6*, *NOX4*, and *TGFBR3*, while 2.5% of SNPs were located in regulatory regions. Biotype classification showed that most variants (93.7%) occurred in protein-coding regions, with smaller proportions in lncRNA, enhancer, and miRNA regions. SNP clustering revealed potential genomic hotspots, with *ST3GAL6* and *DGKZ* exhibiting exceptionally high densities, suggesting roles in FCR-related regulatory pathways. Functional enrichment analysis via g:Profiler identified significant associations with transcription factor binding motifs, including PU.1, NF-E4, and MAFA, as well as enrichment of catalytic activity and phosphatase-related complexes. These findings highlight potential genetic targets, such as *ST3GAL6* and *DGKZ*, and regulatory networks, including catalytic activity (GO:0003824), that may influence FCR.

## 1 Introduction

Feed conversion ratio (FCR) is a key quantitative indicator of production efficiency, defined as the ratio of feed intake to body weight gain over a specific period. It reflects the animal's ability to convert consumed feed into body mass, with lower FCR values indicating greater efficiency. This trait is of substantial economic importance, as feed costs typically account for more than 70% of total production expenses in poultry farming, thereby directly influencing overall profitability.^1^ As such, improving feed efficiency has become one of the major goals in modern poultry breeding programs. Given its biological and economic relevance, FCR has become a central target in genetic improvement programs. The trait exhibits moderate to high heritability, with estimates ranging between 0.36 and 0.45 in various broiler populations,^2^, ^3^ indicating that genetic selection can effectively improve feed efficiency over generations. However, despite this heritability, the complex polygenic nature of FCR, being influenced by numerous genes with small effects, poses challenges for traditional selection approaches. Therefore, identifying the genetic architecture underlying FCR remains a critical step toward improving selection accuracy and understanding its biological basis. Genome-wide association studies (GWAS) have emerged as powerful tools to identify loci and single-nucleotide polymorphisms (SNPs) associated with complex traits like FCR. Numerous QTLs affecting FCR have been mapped in chickens, and many peak SNPs with strong statistical significance have been identified.^4^, ^5^, ^6^ These peak SNPs represent optimal candidates for downstream analysis as they are most likely to reside near or within causative genes. In this study, the term *“SNP clustering”* refers to the accumulation of multiple significant SNPs within a narrow genomic region, which may indicate linkage disequilibrium or selective pressure acting on functionally important loci related to FCR. However, while GWAS can detect associations, functional interpretation of these SNPs requires further analysis to reveal how they affect gene regulation and biological pathways.

To interpret the biological function of these SNPs, functional annotation tools such as the Ensembl Variant Effect Predictor (VEP) are widely used. VEP predicts the molecular consequence of each SNP and its potential impact on gene structure and regulation.^7^ Furthermore, genes identified through SNP mapping can be subjected to functional enrichment and pathway analysis using platforms like g:Profiler, which integrates information from databases such as Gene Ontology (GO), KEGG, Reactome, TRANSFAC, and WikiPathways. These analyses help reveal underlying regulatory pathways and biological processes that link genetic variation to phenotypic traits.^8^

Taken together, these approaches provide a powerful framework to bridge statistical associations with biological meaning. Therefore, the present study aimed to integrate SNP annotation and pathway enrichment analyses to identify key genes, regulatory networks, and molecular pathways associated with FCR in broilers. This integrative workflow, from SNP retrieval to annotation and biological interpretation, offers a clearer understanding of the genomic mechanisms driving feed efficiency in poultry.

## 2 Materials and methods

### 2.1 SNP extraction

An initial set of 2272 peak SNP records associated with feed conversion ratio (FCR) in broiler chickens was retrieved from the Animal Quantitative Trait Loci (QTL) Database (Animal QTLdb^9^;). These records represented significant SNP–trait associations reported in genome-wide association studies (GWAS) of FCR. Because the database-derived dataset contained SNPs reported repeatedly across studies and potentially redundant records, a systematic filtering procedure was applied before functional annotation. First, duplicate and redundant SNP records were removed. To prioritize reproducible association signals and reduce the influence of study-specific findings, SNPs were subsequently retained when they had been reported as significantly associated with FCR (*p* < 1 × 10^−5^) in at least two independent studies. Application of these filtering and deduplication criteria reduced the initial dataset of 2272 peak SNP records to 513 unique, non-redundant SNPs. These 513 SNPs constituted the final analytical dataset and were used for all subsequent functional annotation, gene mapping, SNP-density assessment, and pathway and functional enrichment analyses.

### 2.2 Functional annotation using VEP

The 513 unique SNPs were submitted to the Ensembl Variant Effect Predictor (VEP) tool for functional annotation.^7^ VEP provides detailed predictions of each variant's genomic context and molecular consequence, including annotations such as genomic location, consequence type, predicted impact, biotype, and feature type, based on the latest *Gallus gallus* genome assembly (GRCg7b).This analysis step aimed to determine the potential molecular effects of each SNP, allowing us to classify variants according to their genomic positions (e.g., coding, intronic, or regulatory regions). The resulting annotated dataset was then used as input for downstream pathway and enrichment analyses. All annotation outputs were cross-checked for internal consistency using Ensembl's default quality-control flags, and variants with low confidence annotations (VEP impact = “modifier”) were excluded from enrichment analysis to reduce false-positive associations.

### 2.3 Pathway and functional enrichment analysis

The genes identified through VEP annotation were analyzed for functional enrichment using the g:Profiler web server,^8^ an online bioinformatics tool that identifies enriched Gene Ontology (GO) terms, pathways, and transcription factor motifs among gene sets. The analysis included a wide range of biological databases, such as Gene Ontology (GO) for biological processes, molecular functions, and cellular components; KEGG for metabolic and signaling pathways; Reactome for curated molecular interactions; KEGG for metabolic and signaling pathways and TRANSFAC for transcription factor binding motifs. Integrating multiple databases ensured a broad functional interpretation of the annotated genes. To ensure the robustness of results, statistical significance was evaluated using the Benjamini–Hochberg FDR correction, and only terms with FDR < 0.05 were considered significant. This integrative analytical pipeline, from SNP extraction to annotation and pathway enrichment, was designed to identify the key biological mechanisms and regulatory networks underlying variation in feed efficiency.

## 3 Results

**Fig. 1:**
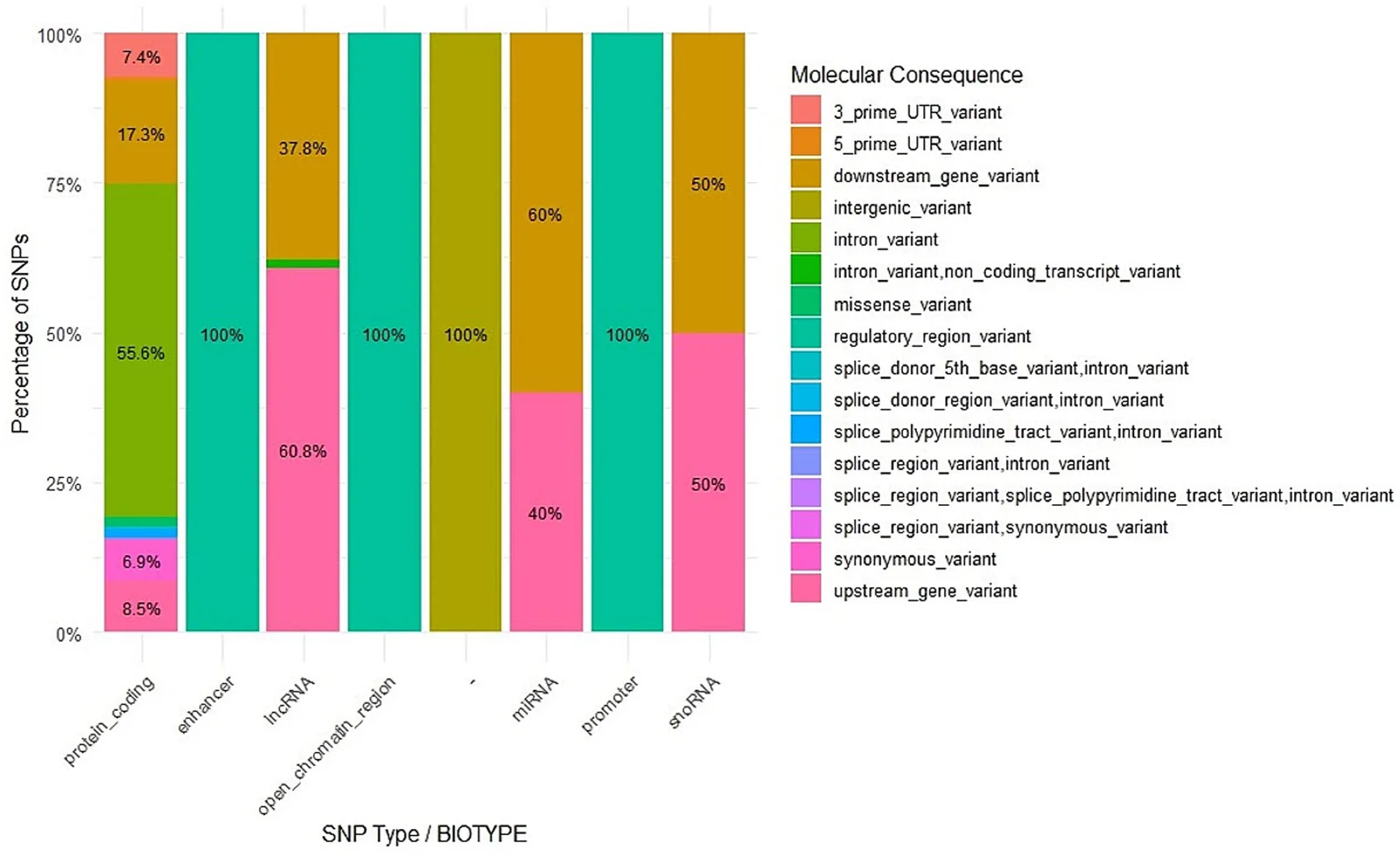
Distribution of SNP types (BIOTYPE) and their molecular consequences in 513 peak SNPs associated with feed conversion ratio (FCR) in Marshall broilers. Each bar represents the proportion of SNPs within a given BIOTYPE, with different colors indicating the type of molecular consequence (e.g., missense, synonymous, intron, UTR, splice variants).

Fig. 1 illustrates the distribution of SNP types (BIOTYPE) and their molecular consequences among the 513 peak SNPs associated with FCR in Marshall broilers. Out of the initial 2272 peak SNPs associated with FCR retrieved from the Animal QTL Database, a subset of 513 unique, non-redundant SNPs was selected for functional annotation. These SNPs were successfully mapped to 543 genes using the VEP, enabling comprehensive downstream analyses of their potential biological roles. This filtering step allowed the focus to remain on the most biologically relevant loci, thereby improving the interpretability of subsequent analyses.

To better understand the functional implications of the identified SNPs, both their molecular consequences, referring to the predicted effects on transcript structure, and their biotype classifications, indicating the nature of the affected genomic element, were thoroughly analyzed. These assessments provided insights into the distribution of SNPs across various genomic regions and their possible functional roles. By separating the results into molecular consequence and biotype classifications, a clearer understanding of how these variants may influence gene function was achieved.

Analysis of the molecular consequences of the 513 curated SNPs revealed that the majority (54.0%) were classified as intron variants, indicating a potential role in modulating gene expression through splicing regulation or non-coding RNA interaction. A subset of SNPs (1.4%) was annotated as missense variants, representing sequence changes predicted to alter the encoded amino acid. These variants were mapped to several genes, including *ST3GAL6*, *NOX4*, *BLM*, *RPL22*, *MVP*, *TXLNB*, *WNT11B*, *MBOAT4*, *ANKRD1*, and *TGFBR3*. Additionally, 6.5% were classified as synonymous variants, which, despite not altering the amino acid sequence, may influence translational efficiency or splicing. A notable group of 1.2% were identified as splice_polypyrimidine_tract_variant, intron_variant, which can affect recognition of the polypyrimidine tract at the 3′ splice site and disrupt normal splicing. These variants were mapped to genes such as *SLC38A2, HMGA2, HAL, CSNK2A2, C12orf65, VPS4B, PAK5, GTF2H5, TMED8,* and *OSTN*. Variants were also found in upstream (10.2%) and downstream (17.7%) gene regions, often involved in transcriptional and post-transcriptional regulatory functions. Furthermore, 6.9% of the SNPs were classified as 3′ UTR variants, which may influence mRNA stability, localization, or translational efficiency. An additional 0.57% were located in the 5′ UTR, potentially affecting transcription initiation or ribosome binding. A total of 13 SNPs (2.5%) were annotated as regulatory_region_variant, with the potential to influence transcription factor binding or enhancer/promoter activity. A smaller fraction (2 SNPs; 0.39%) was classified as splice_region_variant, highlighting their proximity to splice junctions and possible effects on splicing fidelity. Notably, 48 SNPs exhibited multiple molecular consequences, reflecting the complexity of their potential functional roles across diverse genomic contexts. These SNPs were mapped to genes such as *COL6A2, IFNGR1, NAT10, SLC38A2, HMGA2, HAL, CSNK2A2, C12orf65, VPS4B, PAK5, GTF2H5, TMED8, OSTN, PPP1R21, YIPF5,* and*ANGEL2* (Supplementary Table 1).These results collectively highlight that most of the SNPs are likely to play regulatory rather than coding roles, emphasizing their influence on gene expression control mechanisms rather than direct protein alteration.

Biotype classification of the 513 curated SNPs revealed that the vast majority (93.7%) were associated with protein-coding features. Additionally, 3.25% mapped to lncRNA, 1.36% to enhancer elements, 0.66% to open_chromatin_region, 0.44% to miRNA, and 0.13% (3 SNPs) to promoter regions. Moreover, 0.17% (4 SNPs) were located in snoRNA regions, mapped to three genes: *ENSGALG00010023016*, *SNORD61*, and *SNORD73*. This distribution suggests that, although protein-coding regions dominate, a small but meaningful proportion of SNPs are found in regulatory and non-coding elements, indicating multilayered genetic control of feed efficiency.

The annotation results further revealed the presence of overlapping SNPs within specific genes, highlighting the existence of genomic hotspots or regions potentially under selective pressure. As summarized in Table 1, the majority of genes (512) harbored between 1 and 10 SNPs, while 23 genes contained 11 to 20 SNPs. Six genes exhibited SNP accumulation in the range of 21 to 30 SNPs, including *CDK2AP1, COL6A2, KIAA1522, MPDZ, STAU2,* and*VPS4B*, suggesting moderate polymorphism and possible roles in regulatory or structural functions related to feed efficiency. Of particular interest were *DGKZ* and *ST3GAL6*, which exhibited exceptionally high SNP densities, harboring 40 and 50 SNPs, respectively. The enrichment of SNPs in *ST3GAL6* and *DGKZ* suggests their potential roles as major contributors to phenotypic variation in FCR. This high SNP density may reflect their involvement in key regulatory networks or their targeting by selection in broiler breeding programs. The concentration of multiple SNPs in a limited number of genes provides further evidence of specific genomic regions that might have been under evolutionary or artificial selection pressure associated with feed efficiency traits.

**Table 1:** Distribution of genes by the number of overlapping peak SNPs associated with FCR.

Number of SNPs per gene	Number of Genes
1–10 SNPs	512
11–20 SNPs	23
21–30 SNPs	6 (CDK2AP1,COL6A2, KIAA1522,MPDZ,STAU2,VPS4B)
40 SNPs	1 (*DGKZ*)
50 SNPs	1 (*ST3GAL6*)

**Fig. 2:**
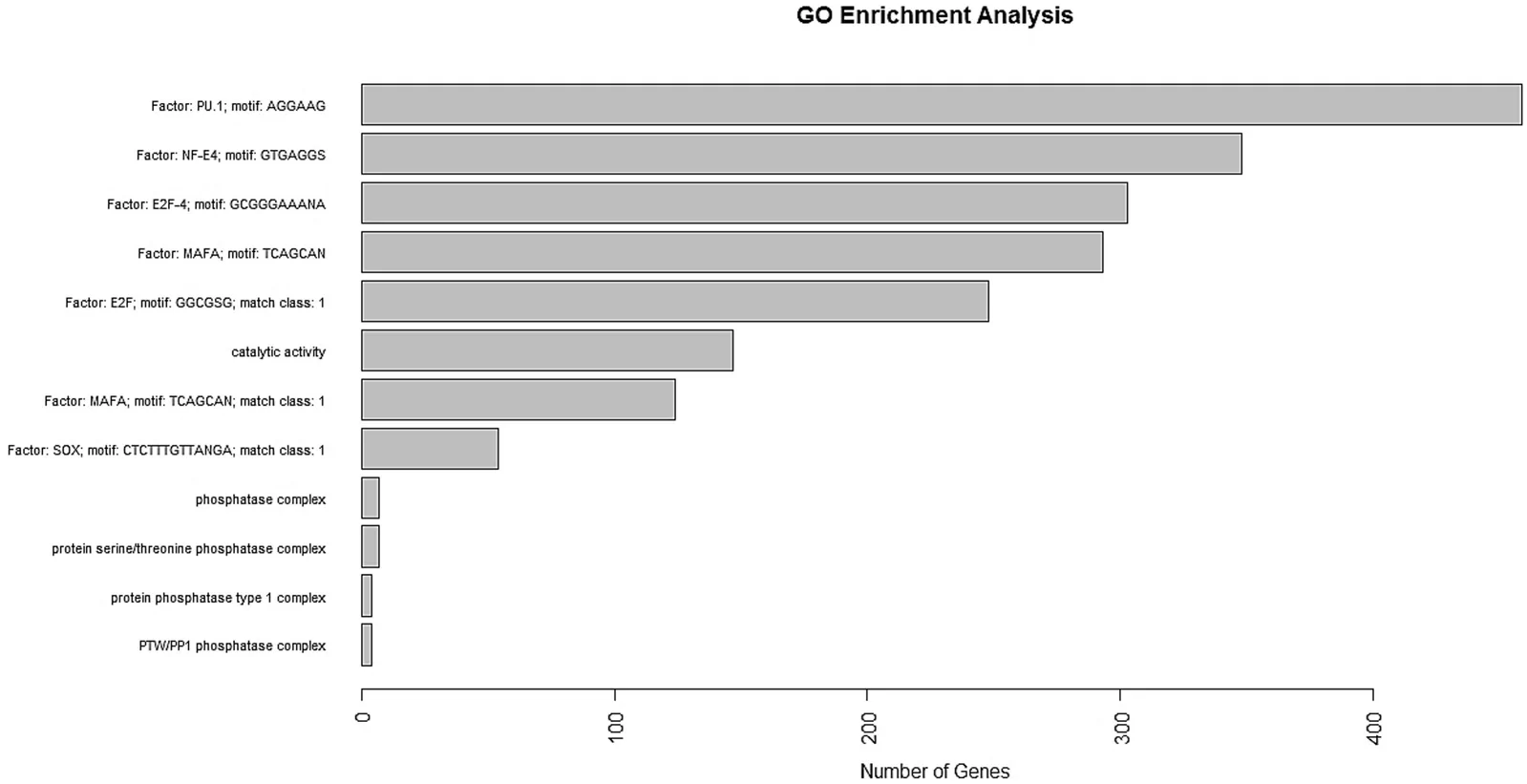
Bar plot representing Gene Ontology (GO) enrichment analysis of genes associated with peak SNPs. The x-axis indicates the number of genes associated with each GO term.

To explore the biological relevance of the 543 genes identified through VEP annotation of 513 unique peak SNPs, pathway enrichment analysis was performed using g:Profiler. This analysis revealed multiple enriched biological processes, molecular functions, and transcription factor binding motifs, reflecting diverse regulatory mechanisms potentially involved in FCR in broiler chickens. Several motifs showed significant enrichment with a high number of associated genes. Notably, the PU.1 (AGGAAG) motif was linked to 459 genes, followed by NF-E4 (GTGAGGS) with 348 genes, E2F-4 (GCGGGAAANA) with 303 genes, E2F (GGCGSG) with 248 genes, and SOX (CTCTTTGTTANGA) with 54 genes. Among the enriched transcription factor binding motifs, a high-confidence match for MAFA (motif: TCAGCAN) was identified under the identifier TF:M01709_1, classified as *match class 1*. This motif was associated with 293 genes, indicating a widespread regulatory footprint potentially involved in metabolic regulation and pancreatic islet development, processes relevant to feed conversion efficiency. Only the high-confidence motif entry (TF:M01709_1) was reported in this analysis (Fig. 2). Although a redundant general entry (TF:M01709) was also detected, it was excluded to avoid duplication, as both refer to the same motif and transcription factor (Supplementary Table 2).These enriched motifs collectively point toward a coordinated transcriptional control involving both general and tissue-specific regulators that influence metabolic pathways linked to feed efficiency.

These findings indicate extensive transcriptional control involving both general and lineage-specific transcription factors. In terms of molecular function, catalytic activity (GO:0003824) was the most significantly enriched term (FDR = 0.0005), involving 147 genes. This strong enrichment suggests a central role for enzymatic processes in determining feed efficiency. Additionally, several phosphatase-related protein complexes were highlighted. The proteinserine/threonine phosphatase complex and the broader phosphatase complex each included sevengenes:*ITPR1**,** PPP1CB**,** PPP1CC**,** PPP1R12A**,** PPP2CA**,** PTPA,* and*WDR82*. More specialized terms, such as the PTW/PP1 phosphatase complex and the protein phosphatase type 1 complex, were enriched by four genes:*PPP1CB***,** *PPP1CC***,** *PPP1R12A*, and *WDR82*.Together, these results demonstrate that enzymatic activity and phosphatase-mediated regulation are central components of the molecular architecture underlying variation in feed conversion ratio.

## 4 Discussion

Our integrated analysis, encompassing GWAS-derived peak SNP selection, VEP annotation, and pathway enrichment, provides novel insight into the genomic architecture underlying FCR in *Marshall* broiler chickens. By combining multiple layers of genomic evidence, this comprehensive approach offers a more holistic understanding of how genetic variation translates into phenotypic efficiency.

The predominance of intronic variants (∼54%) among annotated SNPs aligns with findings that non-coding variants, especially within introns, may influence gene expression through splicing modulation and regulatory element interaction.^10^ The detection of missense variants in genes such as *ST3GAL6*, *NOX4*, and *WNT11B* suggests that, in addition to the predominant regulatory variation, a smaller proportion of FCR-associated variants may exert effects through alterations in protein sequence and function. This interpretation is consistent with previous evidence implicating metabolic and regulatory genes in variation in chicken feed efficiency.^11^ Additionally, splice_polypyrimidine_tract variants (∼1.2%) identified in genes involved in nutrient transport and growth, such as *SLC38A2* and *HMGA2*, may affect splicing efficiency. Although these specific variants have not been widely studied, transcriptomic analyses have shown that splicing regulation plays a critical role in muscle development and feed efficiency in broilers.^10^ Together, these observations suggest that both regulatory and coding variants jointly shape the molecular landscape governing feed efficiency, with intronic variants likely exerting indirect but widespread effects.

In the present study, the vast majority of SNPs (∼93.7%) were located within protein-coding regions, suggesting potential effects on gene regulation or alterations in protein function. A similar trend was observed in chicken GWAS studies, where many significant SNPs associated with feed efficiency and growth traits were reported in or near coding regions, highlighting their functional importance in poultry genetics.^12^This consistency with previous findings reinforces the reliability of our dataset and emphasizes the central role of protein-coding regions in determining efficiency-related phenotypes.

Smaller proportions of SNPs were located in non-coding regions such as lncRNAs**,** enhancer elements**,** miRNAs, and snoRNAs, highlighting the contribution of regulatory non-coding elements to genetic variation in complex traits such as feed efficiency. Similar patterns have been reported in livestock studies, where non-coding RNAs and regulatory elements significantly influence feed efficiency in species like pigs and cattle.^13^Hence, the inclusion of non-coding components in functional annotation expands the biological context, demonstrating that FCR variation arises from both transcriptional and post-transcriptional regulation.

The identification of *ST3GAL6* and *DGKZ* as genes with exceptionally high SNP densities, 50 and 40 SNPs, respectively, marks them as potential genomic hotspots and candidate loci under selection pressure within broiler lines. Such clustering of variants within specific genes hints at their involvement in key metabolic or regulatory pathways affecting FCR. Comparable patterns have been documented in broiler populations, where QTL regions associated with residual feed intake (RFI) and FCR often coincide with regions dense in significant SNPs and candidate gene clusters, including loci like *NSUN3* and *EPHA6.*^14^ These findings underscore the importance of high-SNP-density genes as possible hubs of functional variation, reflecting either adaptive selection or pleiotropic influence on multiple physiological processes.

From an applied perspective, genes such as *ST3GAL6* and *DGKZ* are of high interest for marker-assisted selection or genomic selection programs, as they may carry allelic variation that contributes disproportionately to phenotypic differences. Including dense-SNP genes in custom SNP panels may improve predictive power for feed efficiency traits. Additionally, these loci serve as promising targets for functional validation, via expression analysis or gene editing, to clarify causal mechanisms. Integration with transcriptomic profiles or network analyses could further elucidate the role of these genes in pathways such as glycosylation (in the case of *ST3GAL6*) or kinase-mediated signaling (*DGKZ*). These findings align with previous large-scale broiler GWAS where significant loci were enriched in candidate gene clusters, and selective breeding appeared to concentrate genetic variation in functionally relevant regions.^15^, ^16^ Thus, our observations reinforce the concept that high-SNP-density genes represent focal points in the genomic architecture governing feed efficiency in poultry. Overall, the integration of gene-level and pathway-level insights highlights both specific molecular targets and broader regulatory themes that can guide breeding strategies.

Enrichment analysis identified several transcription factor (TF) motifs, including PU.1, NF-E4, E2F-4, MAFA (high-confidence motif TF:M01709_1, mapped to 293 genes), and SOX. These motifs suggest broad regulatory control over genes potentially involved in FCR. Particularly, MAFA is associated with glucose metabolism and growth regulation, and its regulatory footprint aligns with findings in broiler and indigenous chicken lines where TF-mediated pathways have been implicated in feed efficiency variation.^11^The recurrence of these motifs across numerous target genes suggests coordinated transcriptional regulation that may underlie observed metabolic efficiency differences.

Moreover, the Gene Ontology (GO) term “catalytic activity” (GO:0003824) was the most enriched functional category (FDR = 0.0005), involving 147 genes. This underscores the centrality of enzymatic processes, such as energy metabolism, redox balance, and mitochondrial function, in explaining feed efficiency differences. Transcriptome studies in the duodenum and jejunum of chickens with divergent FCR and residual feed intake (RFI) support this, showing differential expression of metabolism and biosynthesis pathways, mitochondrial genes, and oxidative phosphorylation components.^17^, ^18^This convergence between our genomic results and transcriptomic evidence further supports the idea that metabolic enzyme activity is a key determinant of FCR efficiency.

In our analysis, the Gene Ontology (GO) term **“**catalytic activity**”** (GO:0003824) emerged as the most significantly enriched molecular function (FDR = 0.0005), encompassing 147 associated genes. This enrichment underscores the pivotal role of enzyme-driven biochemical reactions, particularly those related to energy metabolism, redox homeostasis, and nutrient processing, in influencing FCR in broiler chickens. Consistently, proteomic studies in Korat chickens revealed that differentially abundant proteins between high and low feed-efficiency groups were enriched in metabolic pathways such as glycolysis/gluconeogenesis and oxidative phosphorylation, both inherently tied to catalytic function.^19^ Similarly, proteomic profiling of breast muscle in broilers identified mitochondrial enzymes and catalytic proteins as key determinants of feed efficiency variation.^20^ Together, these findings suggest that the genetic architecture shaping FCR is heavily influenced by variation in enzyme-mediated metabolic pathways.

Notably, among the top-enriched terms in our pathway analysis were PTW/PP1 phosphatase complex, protein serine/threonine phosphatase complex, phosphatase complex, and protein phosphatase type 1 complex—all converging on the reversible dephosphorylation machinery centred on PP1 and PP2A family phosphatases. Protein serine/threonine phosphatases are master regulators of cellular signaling and metabolism: by removing phosphate groups from substrate proteins, they modulate enzyme activity, subcellular localization, and signal transduction cascades that control glycogen synthesis, glycolysis, mitochondrial function, and muscle fibre specification (^21^; Ceulemans&Bollen, 2004).Altogether, these findings suggest that phosphatase-mediated regulation acts as a central molecular switch linking energy metabolism with cellular signaling, thereby influencing the overall feed efficiency phenotype in broiler chickens.

## 5 Conclusions

This study identified important genomic regions, genes, and biological pathways associated with FCR in broiler chickens. Functional annotation of 513 SNPs found to be positioned at 543 genes showed that the majority of SNP variants were located in protein-coding regions, and hotspots of SNP density were found in *ST3GAL6* and *DGKZ*. Enrichment studies revealed the catalytic activity and phosphatase complexes, where the key metabolic and signaling processes are catalyzed by enzymes in FCR variation. Collectively, these results can be used to shed new light on the molecular pathways that contribute to feed efficiency and point to genetic targets that can be used in breeding. In a practical sense, the identified genes and pathways are useful genomic selection and precision breeding resources in an attempt to enhance feed efficiency. Incorporating these candidate loci into genomic prediction models or targeted SNP panels may potentially improve the accuracy of selection for feed efficiency in broiler populations. Future research should focus on experimental validation of these candidate genes and regulatory networks using transcriptomic, proteomic, and genome editing. Multi-omics and phenotypic data integration will make causal processes more precise and develop precision-based breeding approaches to improve sustainable poultry production.

## CRediT authorship contribution statement

**Heydar Ghiasi:** Writing – review & editing, Writing – original draft, Visualization, Validation, Supervision, Project administration, Investigation, Formal analysis, Conceptualization. **Navid Ghavi Hossein-Zadeh:** Writing – review & editing, Writing – original draft, Validation.

## Ethical statement

None.

## Funding

None.

## Declaration of competing interest

The authors declare that they have no known competing financial interests or personal relationships that could have appeared to influence the work reported in this paper.

## Data Availability

No data is associated with this manuscript.

## Appendix A Supplementary Data

Supplementary material 1 Detailed functional annotation of feed conversion ratio-associated single-nucleotide polymorphisms in broiler chickens using the Ensembl Variant Effect Predictor (VEP), including genomic location, predicted molecular consequence, impact category, associated gene, feature type, biotype, and transcript or feature identifier.

Supplementary material 2 Results of the g:Profiler functional enrichment analysis of genes mapped to feed conversion ratio-associated single-nucleotide polymorphisms in broiler chickens, including significantly enriched Gene Ontology terms and transcription factor binding motifs, their nominal P-values, false discovery rate (FDR)-adjusted P-values, and associated genes.
